# Antiseptics and Germicides in Dentistry

**Published:** 1898-04

**Authors:** G. S. Martin

**Affiliations:** Toronto Junction, Ont.


AMERICAN JOURNAL

—OF—

DENTAL SCIENCE.

VOL. XXXI. BALTIMORE, APRIL, 1898. NO. 12.

ARTICLE I.

### ANTISEPTICS AND GERMICIDES IN DENTISTRY.

BY G. S. MARTIN, D. D. S., L. D. S., TORONTO JUNCTION, ONT.

Awarded fourth prize (40.00) in "Borolyptol" prize Competition.

It has been said by some one that it is therapeutics that makes dentistry a profession. In no other branch of therapeutics is the dentist so interested as in that given as the subject of this essay: "Antiseptics and Germicides." These terms are commonly used without any distinction being made between them, even writers in our journals and text-books failing often to make any difference.

An antiseptic, as I understand the term, is an agent having the property of preserving organic matter from fermentative changes. A germicide, on the other hand, is an agent that destroys micro-organisms or disease germs. For the most part dentists use germicidal medicaments very much oftener than they do antiseptics, because it is their province to deal with pathological conditions. When a practitioner talks of introducing an antiseptic into an abscess or a putrescent pulp, he generally means a germicide. When he bathes his instruments in a solution it is for the purpose of destroying any lurking germs of disease; and when he cleanses his cuspidore it should be with a germicidal disinfectant. This careless way of speaking is all too common among members of our profession and is very much to be deplored.

In a consideration of the agents at our command I would place first on the list, soap and water, being convinced that clean hands, towel, clothes and instruments are the first essentials to aseptic dentistry. The dentist who neglects cleanliness in his personal habits is not likely to value highly the more specific antiseptics, or to obtain desired results when he does use them. The observing patient is not likely to give a dentist credit for aseptic operations whose finger nails are not even mechanically clean. Dr. Taft has said that a thorough sterilization of instruments may be accomplished by the use of soap and water.

Heat is also one of the most useful of germicides, as, for example, in the cleansing of instruments by a bath in boiling water or oil.

Looking at the list of drugs which are avilable as antiseptics and germicides, we are reminded of the words of the wise king of Israel, regarding the making of books, "there is no end." Probably no other problem so absorbs the best thought of our profession at this one, involving, as it does, the treatment of alveolar abscess, root-canals, pyorrhœa, etc., the greatest trials of a dentist's life. Almost every journal we pick up describes some new or old preparation claimed by some enthusiast to be the ideal antiseptic. A recent writer on pyorrhœa alveolaris counted. if I remember rightly, something over one hundred different remedies for that bugbear of dentistry.

In a paper so limited as this, it will be necessary to apply the principle of exclusion to arrive definitely at the drugs we consider permissible. An ideal antiseptic or germicide should not be escarotic, coagulant, toxic or odorous; should not stain the tissues to which it is applied, and should be possessed of the greatest possible germ destroying power.

On consideration, in the light of these specifications, a great many of the old time favorites will be found wanting in one or more points. For example, such drugs as iodoform and creosote, with their terribly persistent odors are scarcely to be tolerated in a modern dental office. The operating room possesses terrors enough for the delicately organized patient, without the odor of such drugs.

Some of these otherwise desirable medicaments are to be avoided on account of their tendency to stain the tissues; as, for example, iodine or oil of cinnamon in the canal of a prominent tooth will cause a stain very awkward to deal with.

While the question of coagulants and non-coagulants has been a much vexed one during the past year, I am inclined to believe that these agents should preferably be non-coagulant.

Carbolic acid is, perhaps, one of the most common drugs to be found in the average dental office. Pure, it is reliable when applied to an ulcerated surface, or in a cavity before filling a tooth of poor quality. Diluted with water or glycerine, it is an efficient wash in cases of fetid breath from any local cause, such as after extensive extraction. Used one part in twenty of water, it is a reliable bath for instruments. The main objections to its use in the mouth are its odor and its caustic action. The odor may be disguised by the use of oil of cloves.

Carbolic acid, 51 parts, combined with camphor gum, 49 parts, by rubbing in a mortar will give campho-phenique, a remedy which stands to-day one of the most useful in the dentist's cabinet. It is one of the best dressings for root canals, abscesses, etc., and will prevent soreness of separating teeth with cotton. In fact, it has so many uses as to call forth from Professor Flagg, of Philadelphia, the statement that it is "the most remarkable medicament which has ever been offered in connection with dental therapeutics."

Boric acid is an antiseptic possessing very great merit in diseased conditions of the mucous membrane, and after the extraction of a number of teeth. A combination of borax and boric acid, to which some one has given the name boracine, is not caustic, toxic, or irritating, and is tasteless and odorless. A 16 per cent. solution in water has proved excellent in treating abscess of the antrum.

The old-time favorite creosote has, in my practice, given place to less pungent drugs. The only place I ever use it is in combination with tannic acid in cases where I cannot remove all the fibres of a pulp, and even here guiacol is to to be preferred.

The essential oils are valuable where stronger and more irritating agents are not necessary, and because their action is more enduring than watery forms of antiseptics. The most commonly used are cloves, cajeput, cinnamon, eucalyptol and wintergreen.

Bichlorid of mercury is, in spite of the sneers of the men who call it "beg-bug poison," still, perhaps, without a peer as a germicide. The offensive smell may be overcome by the use of a little rosewater. Use 1 part in 1,000 parts of water; it is the best bath for instruments that can be used. Dr. W. D. Miller, after his experiments, placed it at two hundred times stronger than pure carbolic acid, and advises its use even in 2 per cent. solution in cavities before filling. Combined with peroxide of hydrogen its use is indicated in abscess and pyorrhœa. The objections urged against the bichlorid are its poisonous nature, its smell and its tendency to change chemically. The dilute form in which it is used should not, however, result in any harm, and if prepared with ammonia chloride a solution may be made at any time required.

Thymol is a favorite subsitute for creosote and carbolic acid, having, according to most authorities, equal power as an antiseptic without the unpleasant smell. Combined with glycerol it makes a most effective treatment for putrescent conditions of pulps or in any suppurating condition found in the mouth.

One of the most valuable remedies in dental medicine is hydrogen peroxid. Apart from any chemical action on diseased tissues, its purely mechanical services in cleansing by effervesence in contact with pus is of great value to the dentist. Professor Miller places it next to bichlorid in the list of antiseptics. One great point in its favor is the fact that it may be used without fear, as it is harmles to healthy tissues. It should not be injected into an abscess cavity from which there is not plenty of room for the escape of the gases evolved.

Lately, in the treatment of putrescent conditions I have pinned my faith to peroxide of sodium. Dr. Kirk says of this article: "Sodium peroxid is the chemical analogy of the well-known hydrogen peroxid, with several advantages. It is a white solid, strongly alkaline and caustic. It is easily maintained by keeping it from the air, and the solution may be made at any time. It contains thirteen times as much available oxygen as the $H_2O$ of commerce, which can be prepared from it for immediate use by adding to it any dilute acid in proper proportion.

It is, of course, impossible to do more than touch briefly on these agents, and I have selected only those which I use daily in my practice, wishing to make my paper as practical as possible.—*Dominion Dental Journal.*

